# Leveraging Novel Label Design to Improve Older Consumers' Use of Medications

**DOI:** 10.1093/geroni/igaa057.258

**Published:** 2020-12-16

**Authors:** Alyssa Harben, Andrew Nathan, Kaitlin Anderson, Deborah Kashy, Laura Bix, Mark Becker

**Affiliations:** Michigan State University, East Lansing, Michigan, United States

## Abstract

Over-the-counter (OTC) medication is a convenient, affordable way for older adults to treat a variety of conditions; older consumers use more OTCs per capita than other people for varied reasons. As with any medication, OTCs carry risks. Because the labels of OTCs are frequently the only information accessed by consumers, labels designed to communicate safety information are paramount for these products. Yet, available studies suggest that consumers do not regularly access the comprehensive information in the Drug Facts Label (DFL) when making purchase or use decisions, tending to rely on information found on the front of the package. Herein, we evaluate how four OTC label formats (standard; standard/highlight; critical warnings on front/highlighted; critical warnings on front not highlighted) affect how aging participants attend to critical information. Sixty-eight participants (65+) engaged a computer-based task answering yes/no questions that required use of labeling information about the warnings or active ingredients (AI). Results indicate that AI information is found accurately and relatively quickly compared to warning information and highlighting and placement on the front of the package had little effect. By contrast, warning information was found slowly and with low accuracy with the standard label, and highlighting and front of pack placement both significantly improved performance. These results suggest that novel labelling strategies could result in more effective, safer use of OTC medicines among older consumers and provide insights that could be used by regulators as they work to improve policy focused on improving mandates for OTC labeling.

